# A prospective multicenter phase II study of FOLFIRINOX as a first-line treatment for patients with advanced and recurrent biliary tract cancer

**DOI:** 10.1007/s10637-022-01322-7

**Published:** 2022-12-02

**Authors:** Naminatsu Takahara, Yousuke Nakai, Hiroyuki Isayama, Takashi Sasaki, Yuji Morine, Kazuo Watanabe, Makoto Ueno, Tatsuya Ioka, Masashi Kanai, Shunsuke Kondo, Naohiro Okano, Kazuhiko Koike

**Affiliations:** 1 Department of Gastroenterology, Graduate School of Medicine, The University of Tokyo, Tokyo, Japan; 2 Department of Endoscopy and Endoscopic Surgery, Graduate School of Medicine, The University of Tokyo, Tokyo, Japan; 3 Department of Gastroenterology, Graduate School of Medicine, Juntendo University, Tokyo, Japan; 4 Department of Hepato-Biliary-Pancreatic Medicine, Cancer Institute Hospital of Japanese Foundation for Cancer Research, Tokyo, Japan; 5 Department of Surgery, Graduate School of Biomedical Sciences, Tokushima University, Tokushima, Japan; 6 Department of Hepatobiliary and Pancreatic Oncology, National Cancer Center Hospital East, Kashiwa, Japan; 7 Department of Gastroenterology, Hepatobiliary and Pancreatic Medical Oncology Division, Kanagawa Cancer Center, Yokohama, Japan; 8 Oncology Center, Yamaguchi University Hospital, Ube, Japan; 9 Department of Therapeutic Oncology Graduate School of Medicine, Kyoto University, Kyoto, Japan; 10 Department of Hepatobiliary and Pancreatic Oncology, National Cancer Center Hospital Tokyo, Tokyo, Japan; 11 Department of Medical Oncology, Faculty of Medicine, Kyorin University, Tokyo, Japan

**Keywords:** Antineoplastic agents, Biliary tract neoplasms, Cholangiocarcinoma, chemotherapy, FOLFIRINOX

## Abstract

Given the promising activity and tolerability of FOLFIRINOX as a second-line treatment for advanced biliary tract cancer (BTC), it can be an attractive first-line treatment option as well. This is a single-arm, open-label, multicenter phase II study to evaluate the safety and efficacy of FOLFIRINOX as a first-line treatment for patients with advanced BTC. Primary endpoint was progression-free survival (PFS), and the secondary endpoints included overall survival (OS), tumor response and safety. This study defined primary endpoint might be met when the lower limit value of 80% confidence interval [CI] of the median PFS ≥ 6.0 months. Between June 2016 and March 2020, 35 BTC patients (21 intrahepatic, 10 extrahepatic, 2 gallbladder, 2 ampulla) including 26 unresectable and 9 recurrent disease were enrolled. After a median follow-up of 13.9 months, the median PFS and OS were 7.4 (80% CI, 5.5–7.5) and 14.7 (80% CI, 11.8–15.7) months, respectively. Complete response was achieved in 1 (2.9%) and partial response in 10 (28.6%), giving an objective response rate of 31.4% and disease control rate of 74.3%. Major grade 3–4 adverse events included neutropenia (54.3%), leukopenia (34.4%), febrile neutropenia (17.1%), thrombocytopenia (8.6%), cholangitis (8.6%), anemia, nausea, diarrhea, and peripheral sensory neuropathy (2.9% each). FOLFIRINOX was well tolerable in patients with advanced BTC, however, this study did not meet the primary endpoint to conduct a phase III trial. Thus, further explorations are required to find a subset of patients and/or certain clinical scenario which might be beneficial from FOLFIRINOX.

## Introduction

Biliary tract cancer (BTC) comprises heterogenous neoplasms derived from epithelial cells in the biliary system including either intrahepatic or extrahepatic cholangiocarcinoma and cancers of the gallbladder and ampulla of Vater. Although BTC accounts for 3% of all adult cancers, its incidence has been increasing worldwide, primarily due to a rise in intrahepatic cholangiocarcinoma [1]. The prognosis remains dismal, with an all-stage 5-year overall survival of less than 20% [2]. Only surgery can provide a chance to cure but most patients initially present with an advanced disease. Moreover, tumor recurrence frequently develops even after curative surgery. Therefore, palliative chemotherapy plays a crucial role to improve the prognosis of advanced BTC.

A combination therapy of gemcitabine and cisplatin (GC) is a current standard of care for advanced BTC because it provides longer survival without any substantial toxicity as compared with gemcitabine alone [3, 4]. In addition, S-1 is another therapeutic option for BTC [5–7] and GC combined with S-1 (GCS) and gemcitabine and S-1 (GS) has newly added to the first-line treatment based on recent randomized controlled trials demonstrated superiority or non-inferiority to GC [8, 9]. Although several molecular targeted agents combined with or without gemcitabine-based chemotherapy had been failed to show an improvement in survival during the past decade [10–12], novel agents such as fibroblast growth factor receptor inhibitor and isocitrate dehydrogenase-1 inhibitor demonstrated promising results especially for a selected population with specific genome profiles [13, 14]. Recent insights into biological characteristics of BTC might accelerate to establish a novel tailor-made treatment strategy [15], however, it is still warranted to expand therapeutic options with intensification of cytotoxic agents for all BTC patients because targetable molecular alterations are limited in these patients.

Recently, a survival benefit of FOLFOX (fluorouracil, leucovorin, and oxaliplatin) has been shown in patients with BTC refractory to the first-line gemcitabine-based chemotherapy [16]. In addition, liposomal irinotecan combined with fluorouracil and leucovorin has also been contributed to increase survival in the second-line setting [17]. Furthermore, FOLFIRINOX comprises fluorouracil, leucovorin, irinotecan and oxaliplatin has shown activity and tolerability in retrospective and prospective studies for the first- or second-line treatment of advanced BTC [18–24]. Therefore, we designed this prospective study to evaluate safety and efficacy of FOLFIRINOX in patients with chemotherapy-naïve, advanced or recurrent BTC.

## Materials and methods

### Eligibility

Inclusion criteria were as follows: 1) histologically or cytologically proven BTC; 2) advanced or recurrent disease; 3) no prior chemotherapy or radiotherapy except for adjuvant chemotherapy, which had been completed at least 6 months before enrollment; 4) Eastern Cooperative Oncology Group (ECOG) performance status of 0–1; 5) age of 20–75 years; 6) At least one measurable lesion on computed tomography (CT) or magnetic resonance imaging (MRI) defined by Response Evaluation Criteria in Solid Tumors (RECIST) guideline (version 1.1); 7) adequate organ function, as indicated by white blood cell count ≤ 10,000 /mm^3^, absolute neutrophil count ≥ 2,000 /mm^3^, hemoglobin ≥ 9.0 g/dl, platelet count ≥ 100,000 /mm^3^, total bilirubin ≤ 1.2 times the upper limit of normal, aspartate aminotransferase and alanine aminotransferase levels ≤ 2.5 times the upper limit of normal, and creatinine clearance level ≥ 50 ml/min; 8) written informed consent from participants.

The exclusion criteria were as follows: 1) active concomitant malignancy; 2) hypersensitivity to iodine and gadolinium contrast; 3) brain metastasis; 4) blood infusion or hematopoietic growth factor use within 7 days before enrollment; 5) uridine diphosphate glucuronosyl transferase 1 family polypeptide A1 gene (UGT1A1) polymorphisms of homozygous UGT1A1*28 or UGT1A1*6 or heterozygous UGT1A1*6 and UGT1A1*28; 6) marked pleural effusion, or ascites active within 14 days before enrollment; 7) severe comorbidities, such as cardiac, hepatic or renal failure; 8) pulmonary fibrosis or intestinal pneumonitis; 9) grade 2 or higher peripheral sensory neuropathy; 10) active infection other than hepatitis B or C virus infection; 11) user of atazanavir sulfate; 12) pregnant or lactating woman; and 13) disqualified for trial judged by clinical investigators.

### Treatment

We adopted an original, full-dose FOLFIRINOX regimen because at the time of study planning there was little evidence which supported modified regimen such as an omission of bolus-fluorouracil could ensure an improved safety without compromising efficacy of the treatment. Thus, patients initially received oxaliplatin 85 mg/m^2^ over 2 h, irinotecan 180 mg/m^2^ over 90 min, leucovorin 400 mg/m^2^ over 2 h, fluorouracil 400 mg/m^2^ bolus infusion, each once daily on day 1, and fluorouracil 2,400 mg/m^2^ on day 1 over 46 h, every 2 weeks. During the cycle, if patients had a leucocyte > 10,000 ⁄mm^3^, neutrophil < 1,500 ⁄mm^3^, hemoglobin < 8.0 g/dL, platelet < 75,000 ⁄mm^3^, total bilirubin > 1.2 times of the upper limit, AST and ALT > 3 times of the upper limit, creatinine clearance < 50 ml/min, fever > 38℃, grade 3/4 of other non-hematological adverse events, treatment was temporary suspended and the next cycle was resumed after recovery from those toxicities with the reduced doses of each agent including an omission of the bolus fluorouracil infusion. The reduced doses were set at 85 mg/m^2^, 65 mg/m^2^, 50 mg/m^2^, and zero for oxaliplatin, 150 mg/m^2^, 120 mg/m^2^ and zero for irinotecan, and 1800 mg/m^2^ and 1200 mg/m^2^ for continuous fluorouracil infusion. The dose escalation and the use of primary prophylactic granulocyte colony stimulating factor (G-CSF) was not allowed according to the study protocol. The treatment was given until disease progression, unacceptable toxicity, discontinuation as decided by the investigators, or withdrawal of consent.

### Assessment of tumor response and toxicity

The primary endpoint was PFS, and the secondary endpoints included OS, tumor response including objective response rate (ORR) and disease control rate (DCR), and adverse events. PFS was calculated from the start of treatment to the date of either disease progression or death or censored at last follow-up. OS was defined as the time from treatment initiation to final follow-up or until death from any cause. PFS and OS were calculated using the Kaplan–Meier method based on follow-up information, which was received until September 2021. ORR were evaluated with CT every 8 weeks until disease progression or death or censored at last follow-up using RECIST guidelines (ver. 1.1) [25]. Carcinoembryonic antigen (CEA) and carbohydrate antigen 19–9 (CA19-9) were measured at the enrollment and on the day 1 of every 4 weeks. Adverse events were monitored during the study period and graded according to the National Cancer Institute Common Toxicity Criteria (ver. 4.0).

### Statistical analyses

A sample size of 35 patients was calculated to reject a null hypothesis defined as a median PFS of < 6.0 months and accept an alternative hypothesis defined as a median PFS ≥ 10.0 months, with a one-sided significance level of 0.10 and a power of 75%, assuming 5 years of recruitment and an additional 1.5 years of follow-up. In other words, FOLFIRINOX might be considered worthy of further evaluation in a randomized phase III trial if the lower limit value of 80% CI of the median PFS ≥ 6.0 months. All statistical analyses were performed using the program SAS (Statistical Analysis Software 9.4, SAS Institute Inc, Cary, North Carolina, USA).

### Ethical statement

The study was an open-label, multi-center, prospective, single-arm, phase II study to evaluate the safety and efficacy of FOLFIRINOX as a first-line treatment for patients with advanced or recurrent BTC. The study protocol was approved by The Clinical Research Review Board of The University of Tokyo (2018006SP), and all patients provided written informed consent before enrollment. This study was conducted in accordance with the Declaration of Helsinki and the guidelines of Good Clinical Practice. And this study was registered in the University Hospital Medical Information Network (UMIN) Clinical Trial Registry (UMIN000020801) and Japan Registry of Clinical Trials (jRCTs 031,180,082).

## Results

### Patients

Between June 2016 and March 2020, 35 BTC patients were enrolled. All patients had histologically or cytologically proven advanced or recurrent BTC with at least one measurable lesion. Twenty-one patients (60.0%) had intrahepatic cholangiocarcinoma, 10 (28.6%) had extrahepatic cholangiocarcinoma, 2 (5.7%) had gallbladder cancer and 2 (5.7%) had ampulla of Vater cancer (Table 1). Twenty-six patients (74.3%) presented with an advanced disease including 21 (60.0%) metastatic and 5 (8.6%) locally advanced diseases, and 9 (25.7%) had a recurrent disease. The polymorphisms of UGT1A1 were wild-type (*1/*1) in 21 (60.0%) and heterozygous-type in 14 including *1/*28 in 9 (25.7%) and *1/*6 in 5 (14.3%). At the time of data cut-ff on September 2021, 9 patients were still alive but no patients were on the planned treatment of FOLFIRINOX.

**Table 1:** Baseline patient characteristics

		*n* = 35
Age, median (range), years	66	(40–75)
Sex, *n* (%)		
Male	23	(65.7%)
Female	12	(34.3%)
ECOG performance status		
0	27	(77.1%)
1	8	(22.9%)
Location of primary disease, *n* (%)		
Intrahepatic	21	(60.0%)
Hilar	3	(8.6%)
Distal	7	(20.0%)
Gallbladder	2	(5.7%)
Papilla of Vater	2	(5.7%)
Extent of disease, *n* (%)		
Metastatic	21	(60.0%)
Locally advanced	5	(14.3%)
Recurrent	9	(25.7%)
Sites of metastasis, *n* (%)		
Liver	12	(34.3%)
Lung	5	(14.3%)
Lymph node	16	(45.7%)
Peritoneal dissemination	4	(11.4%)
Size of target lesions, median (range), mm	62	(12–176)
CEA, median (range), ng/mL	4.4	(0.5–1457.5)
CA19-9, median (range), IU/mL	113.9	(1.0–103,949)
Biliary drainage, *n* (%)	8	(22.9%)
UGT1A1 polymorphisms, *n* (%)		
Wild type *1/*1	21	(60.0%)
Heterozygous type*1/*6	9	(25.7%)
*1/*28	5	(14.3%)

All values are expressed as *n* (%) or median (range)

*ECOG* Eastern Cooperative Oncology Group, *CEA* carcinoembryonic antigen, *CA19-9* carbohydrate antigen 19–9, *UGT1A1* uridine diphosphate glucuronosyltransferase 1A1

### Treatment exposure

The median number of treatment cycles was 11 (range, 1–63). The median relative dose intensities of oxaliplatin, irinotecan, bolus fluorouracil, infusional fluorouracil, and leucovorin were 62.8%, 60.2%, 13.1%, 76.5%, and 80.0%, respectively. Dose reduction and treatment delay occurred in 30 patients (85.7%). Neutropenia was the most frequent cause for both dose reduction and treatment delay (74.3% and 77.1%, respectively).

### Efficacy

**Fig. 1:**
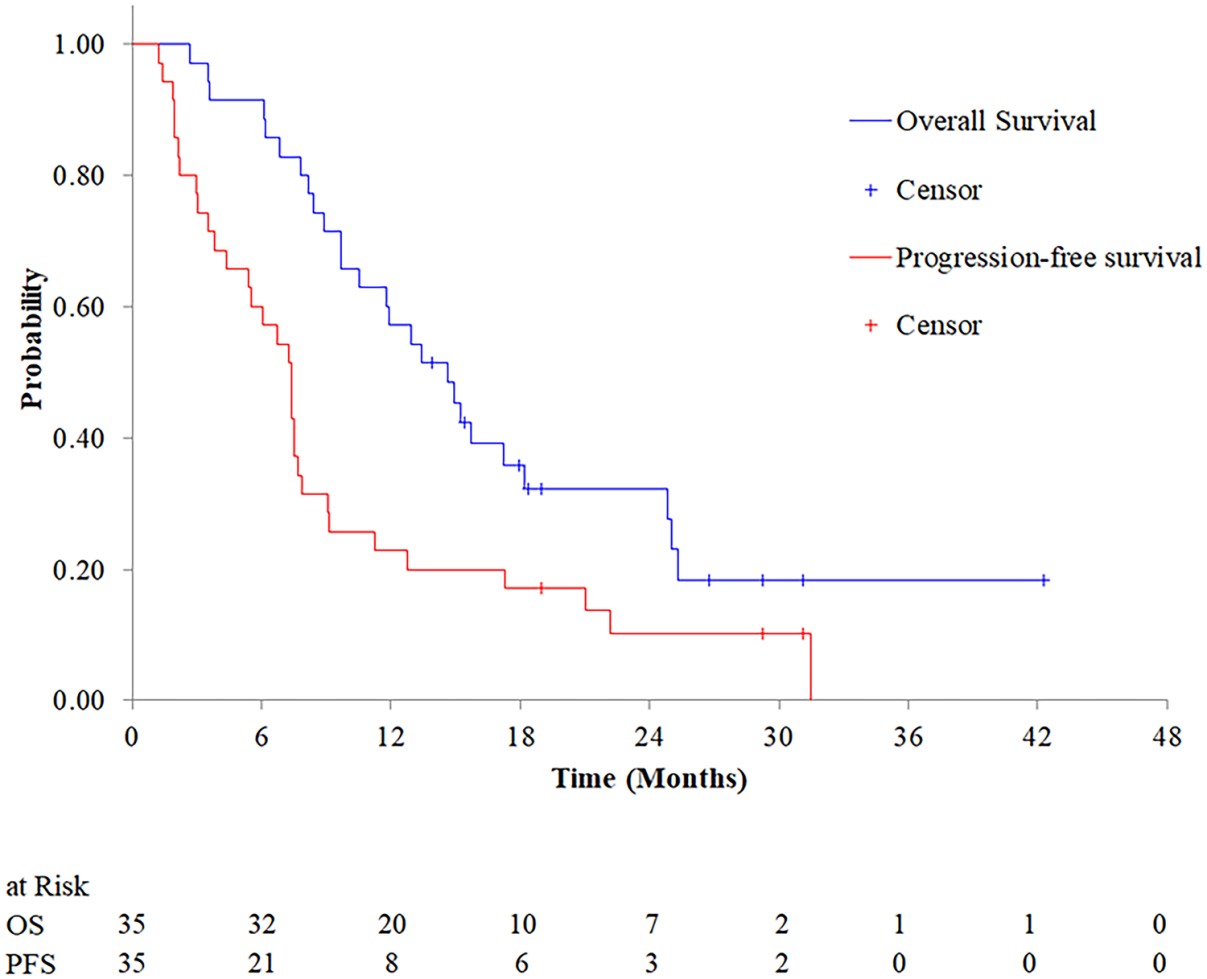
Kaplan–Meier curves for progression-free survival and overall survival. Kaplan–Meier curves for progression-free survival (PFS, red line) and overall survival (OS, blue line). The median PFS and OS were 7.4 (80% confidence interval [CI], 5.5–7.5) and 14.7 (80%CI, 11.8–15.7) months, respectively

After a median follow-up of 13.9 months, the median PFS and OS were 7.4 (80% confidence interval [CI], 5.5–7.5) and 14.7 (80% CI, 11.8–15.7) months, respectively (Fig. 1 and Table 2). The 12-month PFS and OS rate were 12.9 (80%CI, 14.5–32.4) % and 57.1 (80% CI, 45.7–67.0) %, respectively. Since the lower limit value of 80% CI of the median PFS did not exceed 6.0 months, this study failed to achieve the primary endpoint to verify the safety and efficacy of FOLFIRINOX in a phase III trial.

**Table 2:** Efficacy

	*n*	PFS, months	OS, months	ORR, %	DCR, %
All cohort	35	7.4 (5.5–7.5)	14.7 (11.8–15.7)	31.4	74.3
Location of primary disease					
Intrahepatic	21	7.7 (6.7–9.2)	17.2 (13.4–24.8)	28.6	85.7
Hilar	3	7.5 (2.2-NR)	15.7 (2.7-NR)	66.7	66.7
Distal	7	3.5 (2.0–7.5)	9.7 (7.8–14.9)	14.3	57.1
Gallbladder	2	1.7 (1.4-NR)	8.7 (6.8-NR)	0.0	0.0
Papilla of Vater	2	6.4 (5.4-NR)	NR	100.0	100.0
Extent of disease					
Metastatic	21	7.4 (6.0–9.1)	14.7 (11.8–24.8)	28.6	76.2
Locally advanced	5	7.5 (2.0–7.9)	15.7 (3.6–18.2)	40.0	80.0
Recurrent	9	5.4 (2.1–7.4)	12.9 (8.4–15.2)	33.3	66.7
Size of target lesions at baseline					
< 60 mm	17	7.3 (3.8–7.4)	14.9 (9.7–15.7)	35.3	70.6
≥ 60 mm	18	7.5 (5.5–9.1)	14.0 (11.8–18.2)	27.8	77.8
CEA level at baseline					
< 5 ng/mL	19	7.5 (7.4–17.3)	15.7 (13.0–24.9)	42.1	78.9
≥ 5 ng/mL	16	4.1 (3.0–6.7)	10.1 (8.4–14.7)	18.8	68.8
CA19-9 level at baseline					
< 37 IU/mL	11	7.4 (5.4–7.9)	14.9 (12.9–18.2)	36.4	81.8
≥ 37 IU/mL	24	7.3 (4.4–7.5)	13.3 (9.7–15.7)	29.2	70.8

PFS and OS are expressed as median (80% confidence interval) and ORR and DCR are expressed in %

*PFS* progression-free survival, *OS* overall survival, *ORR* objective response rate, *DCR* disease control rate, *NR* not reached

As shown in Table 3, post-hoc analysis showed that PFS was significantly associated with primary cancer and CEA level at baseline, but not with disease extension, tumor size and CA19-9 level at baseline. On the other hand, no factors were significantly associated with OS.

**Table 3:** Factors associated with progression-free survival and overall survival

			PFS				p-value	OS				P-value
			HR	80%CI				HR	80%			
Age	< 65 years	14	1.40	0.87	~	2.24	0.36	1.37	0.83	~ 2.28		0.42
	≥ 65 years	21	Ref					Ref				
Sex	Male	23	0.63	0.39	~	1.03	0.23	0.73	0.43	~ 1.23		0.44
	Female	12	Ref					Ref				
ECOG performance status	0 1	27 8	1.05 Ref	0.61	~	1.83	0.90	0.90 Ref	0.49	~ 1.63		0.82
Location of primary disease	Intrahepatic Hilar	21 3	0.48 0.70	0.18 0.21	~ ~	1.28 2.30	0.34 0.70	0.98 1.16	0.26 0.24	~ 3.77 ~ 5.70		0.99 0.90
	Distal	7	0.79	0.27	~	2.29	0.78	1.96	0.48	~ 7.97		0.54
	Gallbladder	2	24.79	4.06	~ 151.31		0.02	4.18	0.85	~ 20.53		0.25
	Papilla of Vater	2	Ref					Ref				
Extent of disease	Metastatic	21	0.72	0.42	~	1.24	0.44	0.80	0.45	~	1.45	0.63
	Locally advanced	5	0.66	0.30	~	1.45	0.50	0.83	0.37	~	1.85	0.76
	Recurrent	9	Ref					Ref				
Size of target lesions at baseline	< 60 mm ≥ 60 mm	17 18	1.21 Ref	0.76	~	1.93	0.60	0.85 Ref	0.51	~	1.42	0.68
CEA level at baseline	< 5 ng/mL ≥ 5 ng/mL	19 16	0.35 Ref	0.22	~	0.58	< 0.01	0.46 Ref	0.27	~	0.78	0.06
CA19-9 level at baseline	< 37 U/mL ≥ 37 U/mL	11 24	1.02 Ref	0.62	~	1.67	0.97	0.95 Ref	0.55	~	1.65	0.91
Biliary drainage	No	27	0.86	0.49	~	1.50	0.73	0.64	0.35	~	1.18	0.35
	Yes	8	Ref					Ref				

*ECOG* Eastern Cooperative Oncology Group, *CEA* carcinoembryonic antigen, *CA19-9* carbohydrate antigen 19-9, *PFS* progression-free survival, *OS* overall survival, *HR* hazard ratio, *CI* confidence interval, *Ref* reference

**Fig. 2:**
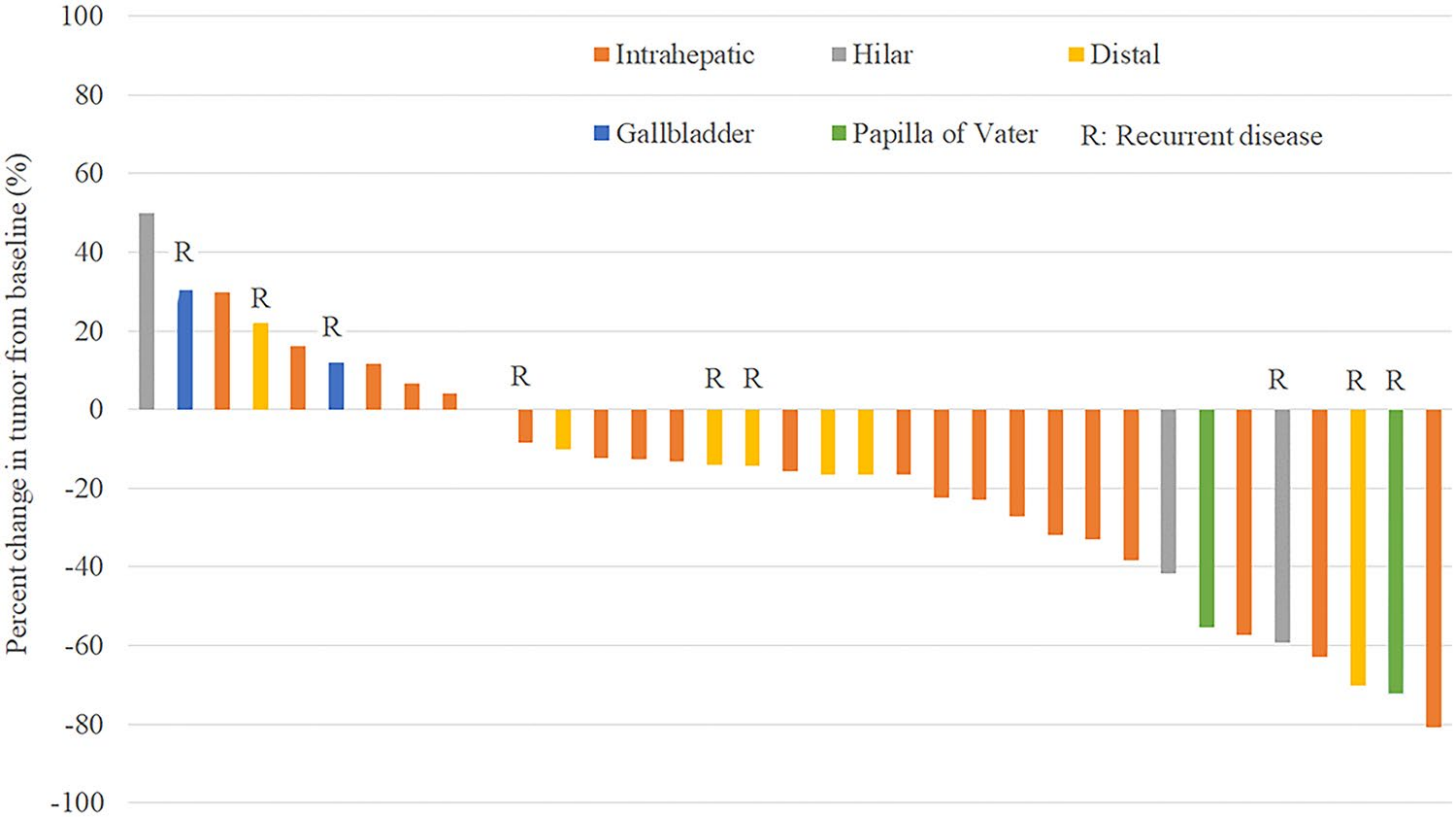
A waterfall plot A waterfall plot shows changes of tumor size at the time of best response from baseline in all 35 patients according to the location of primary disease

A complete response was achieved in 1 (2.9%), partial response in 10 (28.6%) and stable disease in 15 (42.9%), giving an ORR of 31.4% and DCR of 74.3% (Table 2). Best change in tumor volume of the target lesion is shown in Fig. 2. Of note, 8 patients (22.9%) had a remarkable tumor shrinkage by more than 40% from baseline.

Among 24 patients in whom CA19-9 was elevated (> 37 IU/L) at baseline, CA19-9 was reduced by more than 50% compared to pretreatment level in 9 patients (37.5%), which was associated with tumor response (ORR of 71.4% in CA19-9 responder vs. 11.8% in non-responder, data not shown).

### Adverse events

All eligible patients were evaluated for toxicities. Grade 3 or 4 adverse events occurred in 31 patients (88.6%). As shown in Table 4, the major grade 3–4 adverse events included neutropenia (54.3%), leukopenia (34.4%), galactolipid galactosyltransferase increased (20.0%), febrile neutropenia (17.1%), thrombocytopenia (8.6%), cholangitis (8.6%), anemia, nausea, diarrhea, and peripheral sensory neuropathy (2.9% each). There were no treatment-related deaths in this study.

**Table 4:** Adverse events

	Grade 1–4		Grade 3–4	
	*n*	%	*n*	%
Hematological				
Neutropenia	24	68.6%	19	54.3%
Febrile neutropenia	6	17.1%	6	17.1%
Leukopenia	22	62.9%	12	34.3%
Anemia	18	51.4%	1	2.9%
Thrombocytopenia	16	45.7%	3	8.6%
Non-hematological				
Nausea	22	62.9%	1	2.9%
Diarrhea	19	54.3%	1	2.9%
Anorexia	19	54.3%	1	2.9%
Peripheral sensory neuropathy	19	54.3%	1	2.9%
Constipation	15	42.9%	0	0%
Alopecia	11	31.4%	0	0%
Mucostitis oral	10	28.6%	0	0%
Fatigue	10	28.6%	0	0%
Cholinergic syndrome	6	17.1%	0	0%
Biliary tract infection	6	17.1%	3	8.6%
Hiccups	6	17.1%	0	0%
Hypertension	6	17.1%	2	5.7%
AST increased	15	42.9%	1	2.9%
ALT increased	14	40.0%	0	0%
GGT increased	14	40.0%	7	20.0%
Hypoalbuminaemia	12	34.3%	0	0%
ALP increased	9	25.7%	1	2.9%
Hypertension	6	17.1%	2	5.7%
Proteinurea	6	17.1%	0	0%

Adverse events are listed in which grade 1-4 toxicities occurred in more than 15% of patients

*AST* aspartate aminotransferase, *ALT* alanine aminotransferase, *GGT* galactolipid galactosyltransferase, *ALP* alkaline phosphatase

Seven episodes of febrile neutropenia accounted in 6 patients mainly during the first two cycles, and a total of 28 sessions of G-CSF was used in 14 patients (40.0%) to control febrile neutropenia as well as severe neutropenia. The G-CSF usage decreased as the number of cycles increased (12 times during the first cycle, 5 times during the second cycle and 3 times each during the third and fourth cycles). Biliary tract-related events were reported in 8 patients, including cholangitis (*n* = 6), obstructive jaundice (*n* = 1) and biliary tract infection (*n* = 1), and an increased level of blood bilirubin (*n* = 1), all of which were unrelated to the study treatment. For patients with (*n* = 8) or without (*n* = 27) a biliary stent, febrile neutropenia was observed in 3 (37.5%) and 3 (11.1%), respectively and cholangitis was observed in 4 (50.0%) and 2 (7.4%), respectively.

### Post-study treatment

At the time of final follow-up, all patients had discontinued the planned treatment of FOLFIRINOX because of disease progression in 26 (74.3%), unacceptable toxicity in 4 (11.4%; prolonged neutropenia in 2, continuous gastrointestinal bleeding in 1, severe acute respiratory syndrome coronavirus-2 infection in 1), withdrawal of consent in 2 (5.7%), or conversion surgery with curative intent after remarkable tumor shrinkage in 3 (8.6%). Twenty-five patients (71.4%) received second-line chemotherapy (GC in 18, GCS in 4, GC combined with fluorouracil in 2 and S-1 in 1), and 14 patients (40.0%) received third-line chemotherapy (S-1 in 7, GC or gemcitabine in 2 each, GS or clinical trial in 1 each). In total, four patients were converted to be surgical candidates after chemotherapy and they subsequently underwent surgery, resulting in R0 resection in 3. Notably, the surgical specimen revealed a pathologic complete response in one patient with initially metastatic intrahepatic cholangiocarcinoma.

## Discussion

This prospective, multicenter phase II study showed FOLFIRINOX was well tolerable and potentially effective in the first-line treatment of advanced BTC, with a median PFS of 7.4 (80% CI, 5.5–7.5) months and OS of 14.7 (80% CI, 11.8–15.7) months, and an ORR and DCR of 31.4% and 74.3%, respectively. However, since this study did not meet the predefined criterion to conduct a phase III trial (the 80% CI lower limit value of PFS ≥ 6.0 months), further explorations are required to find a subset of patients and/or certain clinical scenario which might be beneficial from FOLFIRINOX.

Since FOLFIRINOX significantly improved the survival in patients with metastatic pancreatic cancer [26], it had been recognized as an attractive regimen worthy of evaluation in BTC. Actually, as shown in Table 5, several retrospective studies reported the promising activity and tolerability of FOLFIRINOX both as first- and second-line treatment, with a median PFS of 5.0–9.9 months, OS of 9.5–15.7 months and an ORR of 16.0%-33.3% [20–22, 24]. Favorable results were also observed in a prospective study focused on gallbladder cancer which generally harbors worse survival compared with other BTC; a median PFS of 8.3 months, OS of 10.2 months and an ORR of 48.2% [19]. However, a recent randomized phase II trial comparing modified FOLFIRINOX and GC (PRODIGE 38 AMEBICA) failed to demonstrate the superiority of FOLFIRINOX over GC in terms of 6-month PFS rate (44.6% vs. 47.3%), and thus concluded GC remains the standard of care for advanced BTC, even though promising results in secondary endpoints; the median PFS of 6.2 months, OS of 11.7 months with an ORR of 25.0% and a DCR of 66.3% with a manageable safety profile [18]. Although inter-study comparison is difficult, our results on the efficacy appeared comparable to or even better than those of previous trials, but as the predefined criterion of PFS was not met, we consider it reasonable not to proceed to a phase III trial comparing FOLFIRNOX with GC without reconsidering the target of patients based on previous trials including ours. In addition to the insufficient activity of FOLFIRINOX, there are some possible reasons for our unsatisfactory study results. Exclusion of patients without measurable lesions could negatively affect the results because this population generally has favorable outcomes. Nonetheless, nearly one third of our patients responded to FOLFIRINOX and we had a few but exceptional responders who reached a remarkable tumor shrinkage, long-term tumor stabilization, or conversion surgery. No obvious factors other than CEA were found in our study to predict clinical benefits from FOLFIRINOX. Previously, two predictive biomarkers for FOLFIRINOX had been identified; microRNA (MIR1307) and Carboxylesterase 2 may play a key role in modulating of chemosensitivity [27, 28]. Additionally, patients harboring DNA damage repair gene mutations including BRCA mutation were reported to have a remarkable response to FOLFIRNOX [29]. Given that treatment options are scarce in patients with BTC, it is essential to explore subsets of patients who might be beneficial from FOLFIRINOX and to further evaluate the safety and efficacy of FOLFIRINOX in the selected patients.

**Table 5:** Previous studies of FOFIRINOX for biliary tract cancer

Author	Year	N	Study design	Setting	Regimen	PFS, months	OS, months	ORR, %	DCR, %
Phelip et al. [18]	2021	92	Randomized phase II	First-line	Modified	6.2	11.7	25.0	66.3
Sharma et al. [19]	2021	29*	Prospective	First-line	Modified	8.4	10.3	48.3	79.3
Ulusakarya et al. [20]	2020	27	Retrospective	First-line	Modified	8.0	15.1	29.3	75.6
Cui et al. [21]	2021	15*	Retrospective	First-line	Modified	5.0	9.5	16.0	76.0
Zou et al. [22]	2021	27	Retrospective	First-line	Modified	9.9	15.7	33.3	77.8
Belkouz et al. [23]	2020	30	Prospective	Salvage	Full-dose	6.2	10.7	10.0	66.7
Ye et al. [24]	2021	15*	Retrospective	Salvage	Modified	6.7	13.2	26.7	80.0
Present study	2022	35	Prospective	First-line	Full-dose	7.4	14.7	31.4	74.3

*PFS* progression-free survival, *OS* overall survival, *ORR* objective response rate, *DCR* disease control rate

* indicates gallbladder cancer

Recent insights into genomic characterization of BTC leads to intensive investigations to develop a target therapy [30]. Rationale for the target therapy for BTC is supported by the MOSCATO-01 trial, which demonstrated an improved survival for those patients who received targeted therapy based on identified molecular findings compared to those treated with unselected therapies (median OS of 17 *vs* 5 months; *p* = 0.008) [31]. Thereafter, several novel options such as FGFR inhibitors and IDH inhibitors had been emerged and provided a dramatic paradigm shift in the treatment for intrahepatic cholangiocarcinoma [13, 14]. In addition, immunotherapy has been actively researched in BTC patients [32, 33]. Most investigations focused on the application of immune checkpoint inhibitors (ICIs) in only a subgroup of BTCs with microsatellite-instability (MSI) high /DNA mismatch repair-deficient (dMMR) or tumor mutational burden (TMB)-high, and limited activity was observed in the second- or subsequent-line settings [34, 35]. However, a recent randomized trial (TOPAZ-1 trial) demonstrated that durvalumab (a PD-L1 inhibitor) combined with GC significantly improved survival and that ICIs-combined chemoimmunotherapy is poised to become a new frontline therapy option, regardless of TMB and MMR/MSI status [36]. With the expanding treatment options, identifying biomarkers to match patients with the highest yield therapies will be critical for maximizing survival for patients with BTC.

FOLFIRINOX for pancreatic cancer is generally reserved to young patients with good performance status and less comorbidity in current clinical practice because of the concerns for safety. To date, modified regimen such as an omission of bolus fluorouracil or initial dose reduction of irinotecan has been recognized as a community-standard in patients with pancreatic cancer since it ensured the efficacy with an improved tolerability [37]. In this study with full-dose original regimen, the major grade 3–4 adverse events were observed in 88.6% of patients including neutropenia (54.3%), febrile neutropenia (17.1%), thrombocytopenia (8.6%) and cholangitis (8.6%) but these adverse events were all manageable with no treatment-related deaths. In PRODIGE 38 AMEBICA study, modified FOLFIRINOX showed the lower incidence of all grade 3–4 adverse events (72.8%) as well as neutropenia (20.7%) than those of our study probably due to the dose modification and the frequent use of primary prophylactic G-CSF. Given the low dose intensity of each agent in our study, modified regimen with G-CSF support can be an alternative to the original full dose FOLFIRINOX.

This study has several limitations. First, our study is not a randomized, controlled trial included heterogenous patients in terms of origin of diseases with a relatively small sample size. Since this likely interferes with the interpretation of results, future studies will need to consider enrolling patients with specific origin of diseases to appreciate more significant results from homogenous populations and guide clinical practice with a disease location-specific approach. Second, our study required longer time to enroll patients than expected due to slow recruitment. During the study period, gene-panel tests using next-generation sequencing have been introduced into clinical practice in Japan, promoting a personalized precision medicine for BTC. Although gene-panel tests will drive a big paradigm shift in the treatment strategy for BTC, patients with actionable mutations or molecular targets are still limited, necessitating further explorations for novel cytotoxic treatment options. Thus, despite its inherent limitations, our study results would provide some evidence to the current treatment options for BTC.

In conclusion, FOLFIRINOX was safe and potentially effective an option for first-line chemotherapy in patients with advanced BTC but further explorations are required to find a subset of patients and/or certain clinical scenario which might be beneficial from FOLFIRINOX.

## Data Availability

The datasets generated and analyzed during the current study are available from the corresponding author on reasonable request.

## Abbreviations

BTC: Biliary tract cancer
FOLFIRINOX: Fluorouracil, leucovorin, irinotecan and oxaliplatin
GC: Gemcitabine and cisplatin
PFS: Progression-free survival
OS: Overall survival
GCS: Gemcitabine, cisplatin and S-1
GC: Gemcitabine and S-1
FOLFOX: Fluorouracil, leucovorin, and oxaliplatin
UMIN: University Hospital Medical Information Network
jRCTs: Japan Registry of Clinical Trials
ECOG: Eastern Cooperative Oncology Group
CT: Computed tomography
MRI: Magnetic resonance imaging
RECIST: Response Evaluation Criteria in Solid Tumors
UGT1A1: Uridine diphosphate glucuronosyl transferase 1 family polypeptide A1 gene
G-CSF: Granulocyte colony stimulating factor
ORR: Objective response rate
DCR: Disease control rate
CEA: Carcinoembryonic antigen
CA19-9: Carbohydrate antigen 19–9
CI: Confidence interval

## Code availability

YES.

## References

[1] Miyakawa S, Ishihara S, Horiguchi A, Takada T, Miyazaki M, Nagakawa T (2009). Biliary tract cancer treatment: 5,584 results from the Biliary Tract Cancer Statistics Registry from 1998 to 2004 in Japan. J Hepatobiliary Pancreat Surg.

[2] DeOliveira ML, Cunningham SC, Cameron JL, Kamangar F, Winter JM, Lillemoe KD (2007). Cholangiocarcinoma: thirty-one-year experience with 564 patients at a single institution. Ann Surg.

[3] Valle J, Wasan H, Palmer DH, Cunningham D, Anthoney A, Maraveyas A (2010). Cisplatin plus gemcitabine versus gemcitabine for biliary tract cancer. N Engl J Med.

[4] Okusaka T, Nakachi K, Fukutomi A, Mizuno N, Ohkawa S, Funakoshi A (2010). Gemcitabine alone or in combination with cisplatin in patients with biliary tract cancer: a comparative multicentre study in Japan. Br J Cancer.

[5] Kanai M, Yoshimura K, Tsumura T, Asada M, Suzuki C, Niimi M (2011). A multi-institution phase II study of gemcitabine/S-1 combination chemotherapy for patients with advanced biliary tract cancer. Cancer Chemother Pharmacol.

[6] Sasaki T, Isayama H, Nakai Y, Ito Y, Yasuda I, Toda N (2013). A randomized phase II study of gemcitabine and S-1 combination therapy versus gemcitabine monotherapy for advanced biliary tract cancer. Cancer Chemother Pharmacol.

[7] Takahara N, Isayama H, Nakai Y, Sasaki T, Ishigaki K, Saito K (2017). Gemcitabine and S-1 versus gemcitabine and cisplatin treatment in patients with advanced biliary tract cancer: a multicenter retrospective study. Invest New Drugs.

[8] Sakai D KM, Kobayashi S, Eguchi H, Baba H, Seo S et al (2018) Randomized phase III study of gemcitabine, cisplatin plus S-1 (GCS) versus gemcitabine, cisplatin (GC) for advanced biliary tract cancer (KHBO1401-MITSUBA). 2018 ESMO Annual Meeting 6150 10.1002/jhbp.1219 PMC10086809 35900311

[9] Morizane C, Okusaka T, Mizusawa J, Katayama H, Ueno M, Ikeda M (2019). Combination gemcitabine plus S-1 versus gemcitabine plus cisplatin for advanced/recurrent biliary tract cancer: the FUGA-BT (JCOG1113) randomized phase III clinical trial. Annals of oncology : official journal of the European Society for Medical Oncology / ESMO.

[10] Lee J, Park SH, Chang HM, Kim JS, Choi HJ, Lee MA (2012). Gemcitabine and oxaliplatin with or without erlotinib in advanced biliary-tract cancer: a multicentre, open-label, randomised, phase 3 study. Lancet Oncol.

[11] Valle JW, Wasan H, Lopes A, Backen AC, Palmer DH, Morris K (2015). Cediranib or placebo in combination with cisplatin and gemcitabine chemotherapy for patients with advanced biliary tract cancer (ABC-03): a randomised phase 2 trial. Lancet Oncol.

[12] Leone F, Marino D, Cereda S, Filippi R, Belli C, Spadi R (2016). Panitumumab in combination with gemcitabine and oxaliplatin does not prolong survival in wild-type KRAS advanced biliary tract cancer: A randomized phase 2 trial (Vecti-BIL study). Cancer.

[13] Abou-Alfa GK, Sahai V, Hollebecque A, Vaccaro G, Melisi D, Al-Rajabi R (2020). Pemigatinib for previously treated, locally advanced or metastatic cholangiocarcinoma: a multicentre, open-label, phase 2 study. Lancet Oncol.

[14] Abou-Alfa GK, Macarulla T, Javle MM, Kelley RK, Lubner SJ, Adeva J (2020). Ivosidenib in IDH1-mutant, chemotherapy-refractory cholangiocarcinoma (ClarIDHy): a multicentre, randomised, double-blind, placebo-controlled, phase 3 study. Lancet Oncol.

[15] Valle JW, Lamarca A, Goyal L, Barriuso J, Zhu AX (2017). New Horizons for Precision Medicine in Biliary Tract Cancers. Cancer Discov.

[16] Lamarca A, Palmer DH, Wasan HS, Ross PJ, Ma YT, Arora A (2021). Second-line FOLFOX chemotherapy versus active symptom control for advanced biliary tract cancer (ABC-06): a phase 3, open-label, randomised, controlled trial. Lancet Oncol.

[17] Yoo C, Kim KP, Jeong JH, Kim I, Kang MJ, Cheon J (2021). Liposomal irinotecan plus fluorouracil and leucovorin versus fluorouracil and leucovorin for metastatic biliary tract cancer after progression on gemcitabine plus cisplatin (NIFTY): a multicentre, open-label, randomised, phase 2b study. Lancet Oncol.

[18] Phelip JM, Desrame J, Edeline J, Barbier E, Terrebonne E, Michel P et al (2021) Modified FOLFIRINOX Versus CISGEM Chemotherapy for Patients With Advanced Biliary Tract Cancer (PRODIGE 38 AMEBICA): A Randomized Phase II Study. J Clin Oncol: Official J Am Soc Clin Oncol JCO2100679 10.1200/JCO.21.00679 34662180

[19] Sharma A, Pramanik R, Kumar A, Pathy S, Kumar S, Bhoriwal S (2021). Safety and Efficacy of Modified FOLFIRINOX in Unresectable or Metastatic Gallbladder Cancer: A Phase II Pilot Study. JCO Glob Oncol.

[20] Ulusakarya A, Karaboue A, Ciacio O, Pittau G, Haydar M, Biondani P (2020). A retrospective study of patient-tailored FOLFIRINOX as a first-line chemotherapy for patients with advanced biliary tract cancer. BMC Cancer.

[21] Cui XY, Li XC, Cui JJ, Wu XS, Zou L, Song XL (2021). Modified FOLFIRINOX for unresectable locally advanced or metastatic gallbladder cancer, a comparison with GEMOX regimen. Hepatobiliary Surg Nutr.

[22] Zou L, Li X, Wu X, Cui J, Cui X, Song X (2021). Modified FOLFIRINOX versus gemcitabine plus oxaliplatin as first-line chemotherapy for patients with locally advanced or metastatic cholangiocarcinoma: a retrospective comparative study. BMC Cancer.

[23] Belkouz A, de Vos-Geelen J, Mathot RAA, Eskens F, van Gulik TM, van Oijen MGH (2020). Efficacy and safety of FOLFIRINOX as salvage treatment in advanced biliary tract cancer: an open-label, single arm, phase 2 trial. Br J Cancer.

[24] Ye LF, Ren C, Bai L, Liang JY, Hu MT, Yang H (2021). Efficacy and safety of modified FOLFIRINOX as salvage therapy for patients with refractory advanced biliary tract cancer: a retrospective study. Invest New Drugs.

[25] Eisenhauer EA, Therasse P, Bogaerts J, Schwartz LH, Sargent D, Ford R et al (2009) New response evaluation criteria in solid tumours: revised RECIST guideline (version 1.1). Eur J Cancer 45(2):228–47 10.1016/j.ejca.2008.10.026 19097774

[26] Conroy T, Desseigne F, Ychou M, Bouche O, Guimbaud R, Becouarn Y (2011). FOLFIRINOX versus gemcitabine for metastatic pancreatic cancer. N Engl J Med.

[27] Carotenuto P, Amato F, Lampis A, Rae C, Hedayat S, Previdi MC (2021). Modulation of pancreatic cancer cell sensitivity to FOLFIRINOX through microRNA-mediated regulation of DNA damage. Nat Commun.

[28] Capello M, Lee M, Wang H, Babel I, Katz MH, Fleming JB et al (2015) Carboxylesterase 2 as a Determinant of Response to Irinotecan and Neoadjuvant FOLFIRINOX Therapy in Pancreatic Ductal Adenocarcinoma. J Natl Cancer Inst 107(8) 10.1093/jnci/djv132 PMC4554193 26025324

[29] Sehdev A, Gbolahan O, Hancock BA, Stanley M, Shahda S, Wan J (2018). Germline and Somatic DNA Damage Repair Gene Mutations and Overall Survival in Metastatic Pancreatic Adenocarcinoma Patients Treated with FOLFIRINOX. Clin Cancer Res.

[30] Nakamura H, Arai Y, Totoki Y, Shirota T, Elzawahry A, Kato M (2015). Genomic spectra of biliary tract cancer. Nat Genet.

[31] Verlingue L, Malka D, Allorant A, Massard C, Ferte C, Lacroix L (2017). Precision medicine for patients with advanced biliary tract cancers: An effective strategy within the prospective MOSCATO-01 trial. Eur J Cancer.

[32] Klein O, Kee D, Nagrial A, Markman B, Underhill C, Michael M (2020). Evaluation of Combination Nivolumab and Ipilimumab Immunotherapy in Patients With Advanced Biliary Tract Cancers: Subgroup Analysis of a Phase 2 Nonrandomized Clinical Trial. JAMA Oncol.

[33] Ueno M, Ikeda M, Morizane C, Kobayashi S, Ohno I, Kondo S (2019). Nivolumab alone or in combination with cisplatin plus gemcitabine in Japanese patients with unresectable or recurrent biliary tract cancer: a non-randomised, multicentre, open-label, phase 1 study. Lancet Gastroenterol Hepatol.

[34] Kim RD, Chung V, Alese OB, El-Rayes BF, Li D, Al-Toubah TE (2020). A Phase 2 Multi-institutional Study of Nivolumab for Patients With Advanced Refractory Biliary Tract Cancer. JAMA Oncol.

[35] Kang J, Jeong JH, Hwang HS, Lee SS, Park DH, Oh DW (2020). Efficacy and Safety of Pembrolizumab in Patients with Refractory Advanced Biliary Tract Cancer: Tumor Proportion Score as a Potential Biomarker for Response. Cancer research and treatment : official journal of Korean Cancer Association.

[36] Oh DY HA, Qin S, Chen LT, Okusaka T, Vogel A (2022) Durvalumab plus Gemcitabine and Cisplatin in Advanced Biliary Tract Cancer. NEJM Evid 1(8). 10.1056/EVIDoa2200015 10.1056/EVIDoa2200015 38319896

[37] Ozaka M, Ishii H, Sato T, Ueno M, Ikeda M, Uesugi K (2018). A phase II study of modified FOLFIRINOX for chemotherapy-naive patients with metastatic pancreatic cancer. Cancer Chemother Pharmacol.

